# Inside the Endometrial Cell Signaling Subway: Mind the Gap(s)

**DOI:** 10.3390/ijms19092477

**Published:** 2018-08-21

**Authors:** Sofia Makieva, Elisa Giacomini, Jessica Ottolina, Ana Maria Sanchez, Enrico Papaleo, Paola Viganò

**Affiliations:** Reproductive Sciences Laboratory, Division of Genetics and Cell Biology, IRCCS San Raffaele Scientific Institute, 20132 Milan, Italy; giacomini.elisa@hsr.it (E.G.); ottolina.jessica@hsr.it (J.O.); sanchez.anamaria@hsr.it (A.M.S.); papaleo.enrico@hsr.it (E.P.); vigano.paola@hsr.it (P.V.)

**Keywords:** endometrial cell, pathway, proliferation, decidualization, migration, angiogenesis, regeneration, breakdown, implantation

## Abstract

Endometrial cells perceive and respond to their microenvironment forming the basis of endometrial homeostasis. Errors in endometrial cell signaling are responsible for a wide spectrum of endometrial pathologies ranging from infertility to cancer. Intensive research over the years has been decoding the sophisticated molecular means by which endometrial cells communicate to each other and with the embryo. The objective of this review is to provide the scientific community with the first overview of key endometrial cell signaling pathways operating throughout the menstrual cycle. On this basis, a comprehensive and critical assessment of the literature was performed to provide the tools for the authorship of this narrative review summarizing the pivotal components and signaling cascades operating during seven endometrial cell fate “routes”: proliferation, decidualization, implantation, migration, breakdown, regeneration, and angiogenesis. Albeit schematically presented as separate transit routes in a subway network and narrated in a distinct fashion, the majority of the time these routes overlap or occur simultaneously within endometrial cells. This review facilitates identification of novel trajectories of research in endometrial cellular communication and signaling. The meticulous study of endometrial signaling pathways potentiates both the discovery of novel therapeutic targets to tackle disease and vanguard fertility approaches.

## 1. Entrance

**Figure 1:**
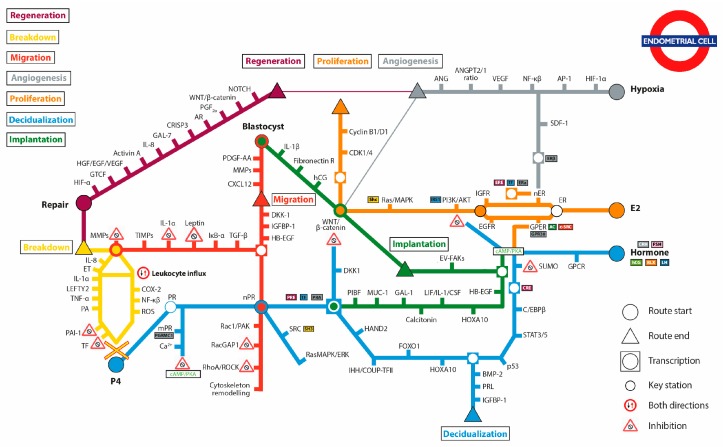
Endometrial cell signaling network illustrated as a subway map showing the seven routes operated by different molecules, narrated in the review. TF in blue boxes denotes transcription factors. All abbreviations are expanded in the main text. The X mark in the red circle indicates progesterone withdrawal.

The compound adjective “highly dynamic” is a cliché when it comes to portraying the endometrium. Nonetheless, it perfectly recapitulates a tissue that quite uniquely executes a remarkable loop of proliferation, differentiation, shedding, and regeneration 400 times in its lifetime. A fine-tuned interplay between ovarian hormones and numerous cell types, including stem and immune cells, governs the orchestration of endometrial cell functions [1]. The tissue itself is stratified into two layers: the functional, a superficial transient layer adjacent to the uterine cavity, and the basal, a deeper permanent layer adjacent to the myometrium. The functional layer consists of a single strand of luminal epithelium, the stroma and the superficial glands (glandular epithelium) whereas the terminal part of the glands is embedded in the basal layer. The thickness of the tissue is determined by its functional layer, which changes throughout the menstrual cycle according to hormonal influences [2]. The phases of the menstrual cycle are defined on the basis of phenomena occurring during the ovarian cycle as the follicular phase (day 0 to day 13), the ovulation (day 14) and the luteal phase (day 15 to day 28). Considering the endometrial cycle phenomena this time round, these phases would rather be the menses (day 0 to day 5), the proliferative phase (day 6 to day 13) and the secretory phase (day 15 to day 28). At the end of menstruation, and until the end of follicular phase (day 6–day 13 of cycle), the rapid construction of the functional layer is governed by **proliferation** of endometrial cells, which grow under estrogenic influence [3]. During this proliferative phase, when estrogen levels are high, the tissue is extensively repaired from the damage caused by menses, the innate immunity is suppressed and growth factor molecules lead cell proliferation. Following ovulation and for the duration of the secretory phase (day 14 to day 28), pituitary hormones and ovarian progesterone (P4) take the estrogen-primed functional layer through extensive differentiation towards **decidualization [4]**. The decidualized endometrium is ready to provide the optimum environment for the **implantation** (day 20 to day 25) of the blastocyst and early growth of the embryo [5]. During this period, a number of signaling cascades stemming from both the blastocyst and the endometrium operate to facilitate apposition, attachment and invasion of the blastocyst but also **migration** of the endometrial stromal cells that move towards the site of implantation to counterbalance the blastocyst-induced tissue remodeling [6]. In the absence of implantation, the corpus luteum absorbs and ceases P4 release. In response to P4 withdrawal, the arteries supplying blood to the functional layer constrict, so that cells in that layer become ischaemic and die. The functional layer undergoes **breakdown** and completely sheds to signify menstruation (day 28–day 5), which is characterized by activation of tissue damage and destruction pathways, vasoconstriction, ischemia, and the high abundance of free radicals and immune cells [7,8]. At the final days of menstruation, simultaneous breakdown and repair will cooperate to allow the endometrium to **regenerate** a new functional layer. The process implicates a number of repair mechanisms, including cell transformation and migration to repopulate the endometrial epithelium, early form of vascular remodeling and progenitor stem cells that reside at the basalis layer, the fountain of youth for regeneration [9,10]. The rise in estradiol (E2) enrolls the surface-regenerated functionalis into continual growth during the phase of proliferation, which is facilitated by intense **angiogenesis** aiming to construct a new vascular network. The newly build vascular network further matures under the influence of P4 during the secretory phase. The aforementioned seven functional “routes” of endometrial cell signaling are depicted in a transit map (Figure 1) with a primary purpose to help “passengers” familiar with endometrial research, or newcomers to the field, to decide on the direction in their research, allow overview of the impressive network of activities occurring inside a unique tissue and, plausibly, identify gaps pending narrowing. Below, each route is elaborated to narrate the key mediators participating endometrial cell signaling.

## 2. Proliferation Route: Building the Functionalis

The increasing mitotic activity seen throughout the endometrial surface/glandular epithelium and stroma, governed by E2, intends to thicken the functional layer in preparation for implantation. The concentration of E2 ranges between 40 pg/mL (end of menses) and 250 pg/mL (before ovulation) [11,12]. A minimum of five days is enough to build a thick layer, however, the proliferative phase is not characterized by a uniform period of endometrial growth. The general consensus is that estrogens exert their effect by modifying gene expression through activation of their nuclear receptors or contributing to growth cascades via nongenomic pathways, which can be receptor-dependent or -independent. Proliferative pathways are active in all cellular types and compartments. Elegant human xenograph experiments in mice have introduced the concept of “interactive proliferation” between the stroma and the epithelium [13]. According to this model, the proliferative response originates in the stroma and feedbacks growth pathways via paracrine signaling in the endometrial epithelium. The predominant estrogen receptor (ER) involved in the transduction of proliferative signals is estrogen receptor alpha (ERα) [14], which is expressed in all endometrial cell types during the proliferative phase and in much higher abundance compared to estrogen receptor beta (ERβ) [15]. Expression of ERβ is higher in the secretory phase of the cycle as a consequence of ERα inhibition by P4, a critical step in itself for the establishment of implantation [15,16]. E2 may also bind to transmembrane G protein-coupled estrogen receptor 1 (GPER), which mediates rapid signaling and is reviewed elsewhere [17]. The diversion of the proliferation route at the ER point, illustrated in Figure 1 at the start of the orange line, is a first critical step upstream all proliferative cascades.

E2-dependent transcription leading up to the synthesis of mitogens is mostly active in the stroma, which communicates in a paracrine manner the response to the epithelial cells [18,19]. Indeed, conditional mutagenesis studies established that stromal-derived ERα is fundamental for directing epithelial cell proliferation, while epithelial ERα is expendable [20]. In a genomic ligand dependent manner, E2 binds nuclear ER (nER) in the cytoplasm and following dimerization, allows for its translocation to the nucleus [21]. The dimer acts as a transcription factor by binding directly estrogen responsive element (ERE) on estrogen responsive genes. Alternatively, E2-nER dimers regulate gene expression independent of ERE but through tethering different transcription factors on mitogen-promoting genes [22]. The result of E2-nER transcription is upregulation of genes involved in the G1 to S progression of cell cycle-Cyclin D1, Cyclin D3, CDK1 and CDK3 are amongst those genes [3,23]. Moreover, E2-nER transcription induces insulin-like growth factor 1 (IGF-1) and mitogen-activated protein kinase (MAPK) pathway related genes [24,25,26]. In a positive feedback, IGF-1 and MAPK cascades are involved in the nongenomic ER-dependent and -independent regulation of E2-driven proliferation [27,28]. In this context, the most well characterized nongenomic model of ER action is mediated through the activation of IGF-1 receptor (IGF-1R). According to the model, cytosolic E2-ERα complexes bind the transmembrane part of IGFR resulting in a bidirectional phosphorylation: IGF-1R phosphorylates ER, which phosphorylates IGF-1R to activate two downstream nongenomic mitogenic signaling pathways: Ras/MAPK and PI3K/Akt [23,29,30]. The first involves the phosphorylation of the adaptor protein Src collagen homologue (Shc) followed by the activation of Ras [31]. The Ras/MAPK pathway contains an elaborate kinase cascade that ultimately enhances the activity of the available transcription factors. The pathway can also induce phosphorylation of nER, which upon dimerization and translocation to the nucleus will initiate transcription of MAPK related genes, notably in an E2-independent manner [32]. ER, total and activated ERK1/2 kinase levels are seemingly comparable in stroma and epithelium of the proliferative endometrium, suggesting pathway activity in both compartments [28]. The PI3K/Akt pathway, on the other hand, results from phosphorylation of the endocytic regulator insulin receptor substrate 1 (IRS-1). Activated IRS-1 interacts with the phosphoinositide 3-kinase (PI3K), to generate phosphatidylinositol 3,4,5-trisphosphate (PIP3). Once generated, the phospholipid PIP3 recruits certain kinases to the plasma membrane including the protein kinase B (PKB)/Akt family of kinases [33]. Activation of Akt in the endometrium phosphorylates a number of downstream targets, which play key roles in cell survival in normal but also in pathological conditions in the endometrium [34,35].

The aforementioned alternative for the E2-initiated proliferation route is to bind the membrane-associated ER to set off nongenomic cascades. The GPER, formerly known as G protein receptor 30 (GPR30), mediates rapid responses in several types including endometrial cells [36,37]. It is located on both the plasma and the endoplasmic reticulum membrane and is in high abundance as expected during the proliferative phase [38]. It is assumed that GPER functions from its location in the plasma membrane. Ligand-activated GPER can trigger two different pathways. The first involves the stimulation of the enzyme adenylate cyclase (AC) to produce cyclic adenosine monophosphate (cAMP), which in turns activates the protein kinase A (PKA) pathway ultimately inducing the recruitment of transcription factors to the promoter of genes with a CRE (cyclic-AMP responsive element) [17,39]. The PKA pathway plays an important role in balancing the proliferative activity of endometrial cells. Specifically, the abundance of cAMP defines whether the transcription will be in favor of proliferation, thus inducing cyclin D/E, or not, in which case the expression of p27Kip1 is instead induced [23]. The endometrial tube map (Figure 1) allows for the observation of the pleiotropic properties of the cAMP/PKA pathway. Indeed, the pathway resembles an interchange subway station serving additionally the decidualization and the implantation routes. One of the important functions of the pathway is to successfully inhibit Akt signaling during decidualization [40]. Indeed, recent studies on infertile women have reported that impaired Akt signaling during proliferation might contribute to endometrial ineptitude to promote relevant cascades en route to decidualization [41].

Besides cAMP/PKA, GPER activates the epidermal growth factor (EGF) receptor (EGFR) to induce a consequent downstream signaling of MAPKs and PI3K. The cascade initiates when the ligand activated-GPER recruits tyrosine-protein kinase c-Src that triggers the release of EGF from the membrane. The latter results in transactivation of EGFR and activation of MAPK and PI3K pathways, as described for the nER-IGFR pathway with induction of proliferation-associated gene expression [42,43].

Another critical operator of endometrial proliferation and growth is the canonical WNT/β-catenin pathway. The pathway functions in endometrial cells in a delicate order, whereby early response to E2 through signaling pathways described above provides the transcriptomic supply for molecules that contribute to the regulation of WNT/β-catenin-mediated late endometrial growth [44]. The cascade involves a destruction complex, which is a complex of proteins consisting of AXIN1-2, β-catenin, adenomatosis polyposis coli (APC), casein kinase (CK1) and glycogen synthase kinase 3 beta (GSK3β) [43]. When no WNT ligands bind the receptor frizzled, the complex assembles and both CK1 and GSK3β phosphorylate β-catenin, which undergoes ubiquitination and proteasomal degradation. However, upon binding of WNT ligands, the activation of disheveled blocks the destruction of the complex and β-catenin accumulates in the cytoplasm and can translocate to the nucleus to interact with members of the TCF/LEF transcription factor family, to regulate the expression of genes associated with proliferation and survival such as cyclin D1 and c-MYC [45,46]. It is believed that the WNT/β-catenin signaling operates with greater intensity in the stroma compared to epithelium, which corresponds to higher abundance of nuclear β-catenin in that cellular compartment [47]. Early proliferative ERα signaling induces the expression of the receptor Frizzled, numerous ligands including WNT4/WNT5a/WNT7a and β-catenin, hence, promotes nuclear localization of β-catenin in epithelium and stroma [48,49,50,51,52]. On the contrary, the pathway inhibitor Dickkopf-related protein 1 (DKK1) is downregulated by ER signaling in the endometrium [53]. ER-mediated PI3K/Akt and Ras/MAPK pathways additionally positively regulate the WNT/β-catenin pathway via inhibition of GSK-3β, which enhances the intracellular stabilization of β-catenin [54]. There is some evidence that the canonical WNT/β-catenin pathway in the mouse endometrium can be activated by E2 in an ER-independent manner. Specifically, E2 can induce the expression of WNT/β-catenin targets in endometrial epithelial cells lacking ER [55]. The authors confirmed this observation in vivo in ERα-lacking mice [56]. Although understanding the mechanism of the ER-independent activation of WNT/β-catenin could help scrutinize endometrial cancer, where the expression of the pathway components is markedly impaired, this area remains unexplored in humans. The subway analogy allows appreciating the importance of WNT/β-catenin system in decidualization, implantation and angiogenesis with some operations in the route towards regeneration. The research into WNT/β-catenin serving migration is also emerging.

A decade ago, the field was introduced to the microRNAs (miRNAs), small noncoding RNAs with posttranscriptional regulation properties. These RNA binding molecules can either degrade mRNAs or suppress their translation. Since their discovery in the physiological and pathological endometrium, miRNAs have been mostly investigated in luteal phase [57,58]. Recently, the first global characterization of miRNAs in the proliferative endometrium emerged to back up individual studies suggesting miRNAs as important players in the fine-tuning of endometrial growth [59]. How different miRNAs regulate components, targets and even transcriptional outcomes of ER-driven signaling in the proliferative endometrium is yet to be fully understood and consolidated but is expected to shape the future of research in the field.

A detailed transcriptomic regulation emanating from ER-mediated E2 operation in the proliferative human endometrium has been systematically reviewed in human and mouse [25]. Better characterization of the operative pathways that induce this transcriptomic signature will generate new targets to circumvent aberrant proliferation that will most definitely lead to failed differentiation [60] and to numerous pathologies including endometrial hyperplasia, cancer, endometriosis and infertility [61,62].

At the end of the proliferative phase after ovulation, the locally rising P4 shifts the endometrium towards a state of endometrial receptivity, a tightly regulated phase in which the endometrium is receptive to embryo implantation.

## 3. Decidualization Route: Priming the Endometrium for Implantation

Decidualization is the process by which P4 induces endometrial stromal cell differentiation into decidual cells to form a new tissue termed decidua. The decidua provides a source of growth factors and cytokines that regulate embryo invasion, support embryo development, modulate immune responses, and support angiogenesis [63]. Priming of the endometrium to become receptive is initiated by E2 but requires the intricately coordinated signaling of E2 and P4 between the luminal and glandular epithelia and the stroma [64]. Each endometrial compartment has a distinct agenda. Stromal cells follow simultaneous proliferation and differentiation. In contrast, epithelial cells cease to proliferate and only differentiate. The stromal cells will stop proliferation and only undergo differentiation into decidual cells at the end of the receptive phase, when already introduced to a blastocyst. From mid-secretory phase, differentiation of stromal cells predominates over proliferation. Usually cellular differentiation follows cell cycle arrest and inhibition of proliferation, however during the secretory phase these functions are temporal. The mechanisms controlling the interconnection of P4 and E2 in the regulation of cell cycle in endometrial cells are surprisingly poorly comprehended, highlighting a major gap in endometrial physiology.

The molecular protagonists in the decidualization route are P4 and cAMP. Because cAMP is involved in routes other than that of decidualization, Figure 1 does not exemplify its cardinal role. A separate branch in the route stemming from cAMP and arriving to the endpoint of decidualization aims, therefore, to signify the independent action of cAMP. Indeed, a spike of LH induces cAMP to elicit an initial and rapid response in endometrial cells while P4 action is independent, slower but persistent. In vitro, the response of endometrial cells to P4 is downstream cAMP activation but this is not believed to be the case in vivo [65]. Nevertheless, it is well established that P4 and cAMP act synergistically to drive endometrial cells through successful decidualization [66]. However, the hierarchy in their responses is still not clear.

At the end of ovulation the endometrium is exposed to high levels of hormones and other endocrine factors such as follicle-stimulating hormone (FSH), relaxin (RLX), corticotropin-releasing hormone (CRH), LH, cyclooxygenase-2 (COX-2) and, in case of pregnancy, human chorionic gonadotropin (hCG) [67,68]. These bind to their respective G protein-coupled receptors (GPCRs) on endometrial stromal cell membrane and stimulate the production of cAMP [69]. The latter will activate the PKA pathway, resulting in phosphorylation of cAMP-response element modulator (CREB), binding to the cAMP-response element (CRE) and initiation of decidualization-specific gene transcription [70]. The genes induced through this pathway include a number of transcription factors capable of interacting with the progesterone receptor (PR) such as forkhead box protein O1 (FOXO1), signal transducer and activator of transcription 5 (STAT5), STAT3 and CCAAT-enhancer-binding protein β (C/EBP β) [67,71,72,73]. In this manner the fast acting cAMP sensitizes stromal cells to the slow-acting P4, which will act through PR in a genomic or nongenomic manner to inhibit epithelial cell proliferation and stimulate differentiation of stromal cells. cAMP is additionally contributing to the cell cycle regulation by inducing the transcription of p53, a tumor suppressor protein, arresting endometrial cells at G2/M checkpoint [74]. Transrepression of p53 from C/EBP β has been observed in endometrial stromal cells with C/EBP β being considered a stabilizer of G2/M inducing factors such as cyclin B2 and CDK1 [75]. Conversely, the other cAMP-induced factor, FOXO1, suppresses cyclin B1/2 and CDK1 [76]. Considering that the cAMP/PKA pathway is an inhibitor of the PI3K/Akt proliferative pathway, the complexity of cell cycle regulation during decidualization is highlighted [40]. An important role of cAMP in sensitizing endometrial cells to P4 is to prevent sumoylation of the PR by altering the expression of numerous small ubiquitin-like modifier (SUMO) enzymes [77]. These downstream targets of cAMP are part of the route branch leading up to decidualization (Figure 1). Recently this branch was reinforced by an interesting study allocating roles for long noncoding RNAs (lncRNAs) in the endometrium [78]. In that work, human decidualization was highly dependent on the expression of the lncRNA LINC473, which was under the positive control of the cAMP/PKA pathway. The downstream targets of LINC473 have yet to be established before its definite roles in decidualization can be confirmed. In light of the recent aspirations to characterize the global lncRNA profile in the endometrium in relation to physiology and pathology, it is envisaged that the gap in our understanding of the RNA binding molecules actions will be eventually filled [79,80,81].

Looking at the tube map illustration, the role of P4 signaling stands strong in the journey towards decidualization. P4, acting in a similar molecular fashion to E2, exerts transcription-dependent and -independent effects in the endometrium. The genomic actions are mediated via the two nuclear progesterone receptors (nPR) subtypes PRA and PRB, upon which P4 binding translocate to the nucleus and associate with progesterone response elements (PRE) in the promoter region of target genes or with other transcription factors and coactivators. PR expression is stimulated by ERα-mediated transcription in endometrial cells and, consequently, E2 is required for P4 responsiveness throughout the luteal phase [82]. Conversely, ERα expression is inhibited by P4 via nPRs [83]. This functional feedback interaction between the two hormonal systems is important for balancing their often-opposing actions. Epithelial cells mostly express PRB, suggesting that PRB is perhaps involved in the control of glandular secretion, whereas PRA is the predominant type in stromal cells and the lack of its expression results in impaired decidualization reflecting the need for prolonged stromal cell PRA-mediated action of P4 in the establishment of pregnancy [84,85]. Different signaling routes have been established for the two receptors. For example, PRB activates rapid cytoplasmic signaling events via interaction with the Src-homology 3 (SH3) domain of the Src tyrosine kinase (SRC) at the plasma membrane, which triggers the Ras/Raf1/MAPK pathway critical for decidualization [86,87]. PRA, on the other hand, is a known transcriptional inducer of differentiation and decidualization. PRA-signaling induces the expression of the basic helix-loop-helix transcription factor (HAND2) in the stroma to suppress the production of fibroblast growth factors (FGFs) and, consequently, their mitogenic action on epithelial cells [88]. In the epithelium, P4 induces the Indian hedgehog (IHH) to activate COUP transcription factor 2 (COUP-TFII) in the stroma [89,90]. Rodent studies showed that COUP-TFII suppresses E2-mediated effects in the epithelium via inhibition of both SRC-1 and ERα phosphorylation [91]. COUP-TFII activates the bone morphogenetic protein 2 (BMP2), which will drive decidualization via activation of the key molecules WNT4 and COX-2.

PR-mediated transcription has profound effects on the WNT/β-catenin pathway [48]. Although the activation of the pathway is critical for implantation, as described later in this review, P4 dramatically upregulates its inhibitor DKK1 in the differentiating stroma and evidently induces blockade of WNT/β-catenin [92]. Albeit repression of the pathway is seemingly essential for proper decidualization, opposing reports add a layer of complexity. For example, WNT4, a potent ligand of the pathway, is increased in the stroma during decidualization in response to the nPR-mediated upregulation of BMP-2 and FOXO1 [93,94]. It is fair to speculate that due to the complexity of the endometrial signaling agenda during decidualization, the WNT/β catenin operates distinctly to meet the needs of each one of the cell functions: proliferation, differentiation, migration and decidualization. It is possible that the pathway is not inhibited during decidualization but reduced to prevent aberrant expression. Indeed, it is thought that embryonic signals stimulate activation of the pathway whereas maternal P4 via DKK1 prevents its hyperactivity to allow for differentiation in the presence of marginal proliferation. Recently, research in the role of miRNAs in the secretory endometrium has identified a novel regulatory pathway by which WNT/β catenin is controlled [95]. The authors of the study observed that P4 induces the expression of miRNA-152, which via direct binding suppresses WNT ligands in endometrial epithelial cells. That study contributed towards delineating the P4-induced suppression of endometrial proliferation in the epithelium. However, more studies are needed to acquire a better understanding of the possible diverse roles of WNT/β catenin pathway en route to decidualization.

The cAMP-induced transcription factor FOXO1 engages in transcriptional cross-talk with the nPR resulting in upregulation not only of the aforementioned WNT4 and BMP-2 but also established markers of decidualization such as the IGFR, IGF binding protein 1 (IGFBP1), prolactin (PRL) and p57 [94]. The nPR-induced transcription factor homeobox protein Hox-A10 (HOXA10) in epithelial cells also contributes to decidualization by elevating stromal expression of IGFBP1, COX-2 and prostaglandin receptors EP3 and EP4 [96]. FOXO1 and HOX10 transcription factors reportedly interact with the nPR on the IGFBP1 promoter [97]. Another cAMP-induced transcription factor, STAT5, which is predominantly expressed in the glandular epithelium with some selective expression in stromal cells, additionally interacts with nPR on the promoter of PRL [98]. The known coactivators promoting the initiation of IGFBP1 and PRL transcription are CBP/p300 and SRC-1/p160, which enhance the activities of the transcription factors in complex with nPR [99,100]. Collectively, PR signaling provides the platform for the formation of a decidua-specific transcriptional complex composed of diverse transcription factors and coactivators leading to the expression of cell cycle regulators (e.g., cyclins, CDKs, p21, p27, p53, p57) or essential decidualizing factors (e.g., BMP-2, PRL, IGFBP1).

Membrane PR (mPR) initiated responses have also been observed with progesterone receptor membrane component 1 (PGRMC1) being the mostly studied in this context [101,102]. Because PGRMC1 was predominantly found expressed in stromal cells as opposed to epithelial cells in the mid-secretory phase, it was initially presumed that it was a critical regulator of decidualization. In the past year, a more convincing study demonstrated that overexpression of PGRMC1 in stromal cells compromised in-vitro-induced decidualization as manifested by attenuated PRL synthesis and absence of typical morphological features [103]. Notably, the authors have previously discovered the PGRMC1 protein to be one of the few differentially expressed between receptive and nonreceptive endometrium [104]. The precise mechanism upstream and downstream mPR activation is yet to be established. However, several studies demonstrated that mPR-induced mobilization of intracellular Ca^2+^ in endometrial cells is known to activate MAPK cascades and inhibit cAMP synthesis [105,106]. The latter could explain how overexpression of PGRMC1 inhibits decidualization. These studies have set the seed and expected to stimulate considerable research to fill our gaps in the understanding of membrane-initiated responses to P4 during the process of decidualization.

Upon arrival of the blastocyst to the uterine cavity, the endometrium starts a cascade reaction to accommodate the needs of the blastocyst during the window of implantation.

## 4. Implantation Route: Accepting the Blastocyst

Implantation-associated signaling pathways are largely influenced by maternal P4 and signals emanating from the blastocyst [107]. PR is expressed throughout the endometrial epithelium before blastocyst implantation but reportedly decreases during implantation; hence the role of PR signaling is to establish endometrial receptivity prior to implantation [108]. For this purpose, P4 blocks E2-driven proliferation in epithelial cells and induces genes that allow the endometrium to respond to the embryo and permit its attachment [109]. Apposition and adhesion of the blastocyst occurs in a chemokine and cytokine enriched microenvironment that is integrin-dependent. Implantation-associated cytokines including leukemia inhibitory factor (LIF), interleukin 1 (IL-1) and colony stimulating factor (CSF) as well as EGFs such as the heparin-binding EGF (HB-EGF) and amphiregulin are under P4 transcriptional control [109,110]. It has been recently demonstrated in mice that upregulation of LIF expression requires the downregulation of PRA in endometrial epithelial cells at the time of receptivity [111]. Surprisingly, this mechanism is yet to be explored in humans.

The hallmark of decidualization is polyploidization and some research has informed on the events underlying the increase in the genome DNA content in decidua cells. For example, HB-EGF binds to the EGFR, the synthesis of which is also maintained by P4, to promote decidual growth and establish polyploidization in the stroma through upregulation of cyclin D3 [112]. Death effector domain-containing protein (DEDD) is essential for polyploidization and is highly expressed in stromal cells during decidualization to arrest the proliferating cell at the G2/M checkpoint [113]. DEDD forms a complex with cyclin D3 to stabilize the cyclin D3/CDK4 and cyclin D3/CDK6 complex to allow further growth [114]. Considering the central role of polyploidization in decidualization, we currently know little about the mechanisms that control it although lively mitochondrial activity is reportedly paramount to allow polyploidization [115].

The blastocyst remains for 72 h in the uterine cavity prior implantation. One of the mechanisms by which P4 prevents premature attachment of the blastocyst, is by a PRA-mediated upregulation of mucin 1 (MUC-1) antiadhesive glycoprotein [116]. P4-induced HOXA10 also plays roles during the window of implantation. Increase in epithelial HOXA10 promotes the expression of αvβ3 and α4β1 integrins and induces formation of apical epithelial projections termed pinopodes critical determinant of blastocyst implantation [109,117]. Integrin αvβ3 is further stimulated by IL-1α and IL-1β secreted by the blastocyst, suggesting an active reciprocal mechanisms between mother and embryo. The importance of these embryo-derived interleukins in the implantation-related cascades in the endometrium has been proposed in the late 1990s, but the notion has been challenged in the recent years [118,119]. Hence, more evidence is needed to understand whether their contribution is pivotal. HOXA10-driven induction of EP3/EP4 and COX-2 is also relevant to implantation and P4-guided secretion of chemokines such as IL-8, membrane cofactor protein 1 (MCP-1), chemokine (C-X-C motif) ligand 1 (CXCL1) and C-X-C chemokine receptor type 4 (CXCR-4) is prerequisite for embryo-endometrial cross-talk during the receptive phase [120]. Another example of this cross-talk is the induction of fibronectin receptor in the blastocyst, which is driven by the PR-regulated secretion of calcitonin from the endometrial stroma [121]. Adhesion and invasion of the semiallogenic implanting blastocyst will introduce an immune challenge to the endometrium. P4 signaling negates the challenge and establishes immunotolerance via the expression of progesterone-induced blocking factor (PIBF) in endometrial cells, which alters the arachidonic acid metabolism, inhibits NK cell activity and promotes a Th2 cytokine response in the stroma-infiltrating leukocytes [122,123,124]. Immunotolerance is further fostered by the induction of IL-10 from tolerogenic dentritic cells, recruited by the P4-driven secretion of galectin-1 (GAL-1) from endometrial cells [125].

As mentioned above, active WNT/β-catenin signaling is needed in the process of implantation [126]. Mouse implantation sites are rich in various WNT ligands and receptors and the activity of the pathway itself is greatly increased during the window of implantation in specific endometrial regions close to the invading blastocyst [127,128]. The importance of the pathway is clarified by the impact of its inhibition; pre-treatment of mouse blastocysts with a WNT/β-catenin inhibitors Sfrp2 or Dkk1 results in dramatic decrease in implantation rate [127,129]. The mechanisms underlying the dependence of implantation from WNT/β-catenin are not understood. Theories regarding the possible influence of the pathway on migratory cascades in endometrial cells can be postulated and are discussed later in this review. To date, only one study in mouse has proposed a role for WNT/β-catenin in polyploidization of decidua cells [130]. This notion is both interesting and credible considering the novel discovery that WNT signaling can influence the position and orientation of the mitotic spindle during cell division in other systems [131]. This line of investigation undoubtedly deserves elaboration.

The window of endometrial receptivity has been extensively studied in order to establish a transcriptomic signature compatible with successful implantation and unravel the signaling pathways pursuing it. A recent analysis defined a meta-signature of endometrial receptivity involving 57 transcripts as putative receptivity markers [132]. The meta-signature genes highlighted the importance of signaling with regard to immune responses, the complement cascade pathway and extracellular vesicle (EV)-mediated communication in mid-secretory endometrial functions. These genes and the involved pathways will generate new hypotheses and direct future research to delineate further endometrial cell signaling events during the window of implantation. Some research has already shed light into the utilization of EV trafficking by endometrial cells at the time of implantation. Human endometrial-derived EVs are rapidly internalized by trophoblast cells and enhance their adhesive capacity [133]. The mechanism underlying this functional effect of endometrial EVs is believed to involve the delivery of a cargo rich in adhesion molecules. These include the integrin-binding fibronectin and numerous members of the Focal adhesion kinase (FAK) pathway, all of which increase in trophoblasts following endometrial-EV uptake [133]. The invasion of the blastocyst into the decidua will send the endometrial cells onto a migratory route whereby differentiating stromal cells actively promote implantation by moving around and encapsulating the blastocyst.

## 5. Migration Route: Promotion of Blastocyst Invasion

A function largely neglected by the literature is the migration of endometrial stromal cells during implantation, which is regulated by both the invading blastocyst and the stroma. The embryo itself has a crucial function in modulating stromal gene expression and function to allow for its invasion. An in vitro implantation model whereby human blastocysts were placed on a monolayer of decidualizing endometrial stromal cells showed that within a period of 48 h highly motile cells surrounded the blastocyst [134]. When the motility of cells was suppressed, trophoblast invasion was inhibited. In another model using spheroids instead of blastocysts, decidualizing stromal cells aligned around the spheroid in a different manner compared to nondecidualizing cells, highlighting that cell migration was directed by decidualization [135]. Indeed, in vitro motility was enhanced in decidualizing compared with undifferentiated endometrial stromal cells and both invasion and chemotactic migration largely increased when decidualizing cells were in contact with trophoblasts [136,137]. A recent study refined these observations by exploring how migration is impacted following co-incubation of decidualized and not decidualized cells with secretome of human embryos with different qualitative features [138]. Their classical migration assays confirmed that only good quality embryos stimulate migration of decidualized cells, but notably not of not decidualized cells. A molecular mechanism to account for this observation was not discussed by the authors. However, it is not unlikely that the WNT signaling is partly involved due to its putative role in cell migration in different tissues (reviewed in [131]).

The pleiotropic functions of WNT pathway activation in the endometrial cells makes it extremely difficult to study isolated events, such as migration, and interpret the generated findings. The different modes of WNT signaling—canonical or noncanonical—add an additional layer of complexity. It needs to be emphasized that the research in the field of noncanonical WNT pathway operating in the endometrial cell has barely scratched the surface. Especially of the WNT/planar cell polarity (PCP) signaling pathway that controls tissue polarity and cell movement through the activation of Rho GTPases. Rho GTPases are putative targets of nPR signaling in the endometrium during the window of implantation being a family of proteins that modulate cytoskeleton dynamics, myosin activity and cell adhesion. Rac-1 is a member of the Rho family of GTPases that acts through interaction with p21-activating kinase (PAK). Rac-1-induces promotion of lamellipodial protrusion at the front of migrating cells to provide integrin-mediated adhesion while RhoA induces retraction at the rear [139]. ROCK1 activation by the RhoA generates contractile forces through actin-myosin interactions. Contraction and detachment of trailing edges allows for the promotion of the cell body. Rac-1 reduces RhoA activation, and the RhoA target Rho-kinase (ROCK) can inhibit Rac-1 [140]. P4 sets off rapid nongenomic activation of RhoA/ROCK and Rac-1/PAK cascades that help migration of cells through regulation of cytoskeletal fluidity and continuous destabilization and stabilization of cortical actin stress fibers. Silencing of Rac-1 in human endometrial stroma leads to inhibition of implantation whereas silencing of RhoA results in outgrowth of blastocysts [134,141]. In line, migration of endometrial stromal cells can be directly inhibited by decreasing the activity of ROCK [30]. It is, therefore, well-understood that enhanced endometrial stromal cell motility occurs in the presence of ROCK inhibition, downstream of RhoA.

The link between WNT pathway and RhoA/ROCK has never been explored in the endometrium in this context. However, the ligand mostly associated with noncanonical activation of the WNT receptors is WNT4, which is highly secreted by trophoblasts during implantation [93]. The production and synthesis of WNTs from the invading embryo at a first glance does not fit well with the established inhibition of WNT/β-catenin by P4 signaling in endometrial cells during implantation. Especially because the P4-induced expression of DKK1 inhibitor of the WNT pathway in endometrial cells has been demonstrated to diminish trophoblast invasion [142]. This inconsistency could perhaps be explained by the great gap in the understanding of dynamics in the operation of WNT canonical and noncanonical pathways in endometrium during the window of implantation. It is possible that a canonical-transcription dependent pathway is blocked by P4 to decrease mitosis in the epithelium and at the same time noncanonical-transcription independent pathway, facilitated by Rac1 and ROCK, is underway to promote endometrial cell motility. The plausibility of this notion requires deliberation and has the potential to inform on novel unprecedented aspects of WNT signaling in the endometrium. Due to its unconfirmed status, the migration route in Figure 1 is not crossing the interchange station denoting the WNT pathway.

Endometrial P4-induced IGFBP-1 also contributes to trophoblast migration by activation of α5β1 integrins on the surface of trophoblasts leading to activation of FAK and MAPK cascades [143]. On the blastocyst side, the platelet-derived growth factor AA homodimer (PDGF-AA) is an important putative signal that mobilizes stromal cells at the implantation site [137]. A transcriptomic analysis performed to identify factors with key roles in orchestration of migration during implantation found PDGF-AA to be expressed in competent blastocysts with their corresponding receptor being expressed in the receptive endometrium [144]. PDGF-BB homodimer is also a stimulus of chemokinesis and chemotaxis in undifferentiated and decidualizing cells [137]. PDGF-BB homodimer binding can activate Rac-1 in stromal cells and indirectly inhibit ROCK contributing to enhanced motility [145]. RacGAP1, a GTPase-activating protein that exerts its GAP activity on RhoA and on Rac-1, is downregulated in endometrial stromal cells in response to blastocyst signals and current knowledge suggest that RacGAP1 is upstream of Rac-1 [141]. However, the specific embryo-derived factors mediating the observed reduction in RacGAP1 levels remain to be characterized. Trophoblast cell-derived CXCL12 may be another important factor to stimulate migration and was shown to up-regulate CXCR4 (the receptor for CXCL12) in first-trimester decidual cells and to promote their invasiveness [146]. Additionally, HB-EGF, a multifunctional mediator of embryo-endometrial communication during implantation, is an important chemoattractant for stromal cells acting through EGFR to facilitate endometrial cell migration [137]. CD82, a metastasis suppressor that is specifically induced in the decidual stroma, may have a key role in trophoblast invasion as CD82-positive decidualized stromal cells are highly responsive to trophoblast signals in migration and invasion assays [136,147]. Silencing of CD82 in decidualizing stromal cells results in attenuation of chemotactic migration [148].

Active remodeling of the extracellular matrix (ECM) is a known contributor to the regulation of decidua migration and deep trophoblast invasion leading up to the formation of a haemochorial placenta. Proteolytic enzymes such as matrix metalloproteinases (MMPs) control the invasive growth of trophoblasts and their activity is under negative regulation by P4. The role of P4 demonstrates that the process of ECM remodeling during migration involves both signals that promote it and those that restrict it. In particular, it is believed that P4 functions to prevent excessive invasion [121]. Matrix metalloproteinase-2 and -9 that digest the main component of basal membranes collagen IV are highly secreted by invasive trophoblast cells [121]. P4 blocks the secretion of MMP-9 from trophoblasts and inhibits the activities of MMP-1, -2, -3, -7 and -9 in human endometrial explants where it increases the MMP tissue inhibitor (TIMP)-3 [149,150]. The mechanisms by which P4 affects these factors involve direct transcriptional modulation. P4 inhibits the binding of transcription factor SP4 to the promoter of MMP-2 by directing SP4 degradation and the binding of NF-κB to the promoter of MMP-1, -3 and -9 by upregulating its inhibitor IkBα [151,152]. These events result in overall decrease in MMPs activity. P4 evidently also inhibits IL-1α-induced MMP-3 activation and stimulates TGF-β in stromal cells [153], which activates TIMPs and inhibits MMP-7 expression in the epithelium [154]. The expression of leptin, a P4-regulated gene, is suppressed in endometrium during migration, additionally impacting the availability of MMP-2 and MMP-9 [155].

Vanguard research in the field is slowly introducing a new concept in the regulation of endometrial cell migration: vesicle-mediated communication between endometrial cells and trophoblasts to promote cell motility. Endometrial epithelial cells release EVs containing the glycosylated transmembrane protein extracellular matrix metalloproteinase inducer (EMMPRIN), and this release is increased when cells are stimulated with a GPER ligand [156]. EMMPRIN mediates cell invasion and can induce the release of MMP-9 from endometrial fibroblast [157]. Whether EV-EMMPRIN can act on trophectoderm cells or on neighboring endometrial epithelial cells to contribute to invasion and migration has yet to be explored. In support of the role of EVs in the mechanisms regulating migration, endometrial stromal cell Rac-1 pathway seems to elevate vesicular trafficking [158]. Considering the recent meta-analysis pointing out that numerous genes contained within the human uterine fluid during the secretory phase are involved in vesicle trafficking, the concept of EV-mediated migration of endometrial cells during implantation deserves attention and is set to create a new research trajectory [132]. Decoding the players involved in migration potentiates discovery of candidate therapeutic targets for the management of implantation pathologies.

In the absence of implantation at the late secretory phase, the availability of both steroids falls due to corpus luteum regression. The latter triggers infiltration of leukocytes, proteolytic breakdown, shedding of the endometrium, and consequently menstrual bleeding.

## 6. Breakdown Route: Shedding the Functionalis

Menstrual breakdown is limited to humans, primates and a few mammals including some bats. It results from P4 withdrawal in decidualized stromal cells, which in the absence of PR signaling undergo functionalis-specific tissue degradation to demolish the decidualization-induced assembly of pericellular structures. Complex cascades involving endocrine and paracrine signaling within the endometrium govern the process of shedding. The PR withdrawal-initiated breakdown route (Figure 1) can enhance inflammatory reactive oxygen species (ROS) via inhibition of superoxide dismutase activity, which in turn upregulates NF-κB and COX-2 signaling and results in the production of inflammatory factors, including prostaglandin F2α (PGF2α) [159,160]. PGF2α induces myometrial contractions and vasoconstriction of the spiral arteries both of which are critical events in the menstruation process. However, ROS-mediated activation of NF-κB alone may result in the production of the inflammatory factors such as MCP-1, IL-6, TNF, and IL-1 [161]. These can stimulate influx of neutrophils in the stroma, which also represent a major source of ROS [162]. Stromal cells is an additional source of ROS, which are generated as byproducts of normal metabolism. Perhaps the best-characterized function of infiltrating neutrophils at the time of menstruation is to provide the matrix with proteases such as MMPs [163]. MMPs play a leading role in the breakdown of the ECM during menstruation, which can be reversed by synthetic inhibitors of MMPs [164,165]. Most MMPs are expressed in the human endometrium where their activity is tightly regulated both spatially and temporally to ensure that extensive tissue breakdown is restrained to the functionalis while allowing ECM remodeling during blastocyst implantation. The regulation of MMPs occurs at the levels of transcription, activation, membrane recruitment, TIMP-induced inhibition and endocytic clearance.

Several cytokines/growth factors and other molecules regulate the expression and activity of MMPs in the human endometrium. The most important and well-established of these have been illustrated as stations in a second branch stemming from P4 withdrawal in the breakdown route of Figure 1. One of them is plasmin, detected in high amounts in the menstrual material and generated by plasminogen activators (PAs), which are produced in the endometrium [166]. Plasmin can degrade numerous connective tissue proteins, for example fibronectin, laminin, proteoglycans, and collagen type IV , I.V. Plasmin also activates cytokines in the TGF-β family, which are highly expressed in the human endometrium during menstruation and localized within the stromal cells, glandular cells and macrophages, and found in the shed endometrial tissue [167,168]. Plasmin expression is regulated by P4. During the secretory phase of the cycle, P4 stimulates the expression of the PA inhibitor (PAI)-1 by endometrial stromal cells, leading to an increase in the number of urokinase (uPA) receptors and enhancing internalization of uPA/PAI-1 complexes [169]. At the end of the secretory phase, the low availability of P4 removes the repression of PA activity, enhances the fibrinolytic activity of the menstrual fluid and promotes the degradation of ECM [170]. Another molecule able to stimulate the expression of MMP-1 and MMP-3 by stromal cells is IL-1α [171]. Expression of IL-1α by stromal and epithelial cells is differentially modulated by P4, which inhibits IL-1α in stromal cells via an unknown mechanism but has no effect on epithelial IL-1α [171]. LEFTY-2 (endometrial bleeding associated factor), a member of the TGF-β superfamily, is selectively expressed in the menstruating endometrium [172]. Its expression is strongly repressed by P4 and recombinant LEFTY-2 stimulates the expression of MMP-3, -7 and -9 [173,174]. It is the most potent inducer of MMPs in endometrial cells upon P4 withdrawal at menstruation.

The expression of the potent vasoconstrictor endothelin (ET) reaches a peak in glandular cells during the perimenstrual phase and both TGF-β1 and IL-1α induce its expression [175]. ET receptor B is also upregulated in the stromal and glandular cells at menstruation and its stimulation increases MMP-1 and MMP-3 [175,176]. TNF-α, which is expressed in the wall of the spiral arterioles and in glands at menses, also induces MMP-1, -3, and -9 and mediates apoptosis, cell-cell dissociation in endometrial epithelial cells and compromises vascular integrity leading to haemorrhage [177]. EMMPRIN, EGF, PDGF-BB, IGF-II, CCL-16, CCL-21, IL-8, and IL-6 all contribute to the abundance of MMPs in the stroma [178,179].

The decline in circulating P4 additionally triggers reduction in tissue factor (TF) to create a pro-hemorrhagic and fibrinolytic milieu [180]. TF gene promoter lacks a PRE site, hence its induction by PR in human endometrial stromal cells occurs via enhanced expression of the transcription factor, SP1 and requires the presence of EGF [181]. P4-stimulation of TF expression continues in stromal cells throughout pregnancy to protect against bleeding and possibly contributes to peripartum hemostasis [182].

Although P4 withdrawal is the primary trigger for endometrial breakdown and shedding, the downstream regulators of this signaling are vaguely understood. Scrutinizing the molecular mechanisms has the potential to inform on the pathophysiology of many disorders including heavy menstrual bleeding and postpartum hemorrhage, and therein aid the development of therapeutics for their management.

Menstruation is followed by restoration of vascular integrity, angiogenesis, and efficient endometrial repair [7].

## 7. Regeneration: Repairing the Functionalis

Regeneration of the functionalis occurs simultaneously with degeneration. As early as day 2 of the cycle, during active shedding, stumps of residual glands in the basalis protrude from the stroma forming glandular cones. Glandular epithelial cells proliferate and migrate laterally to repopulate the luminal epithelium in a process termed re-epithelialization [9]. Furthermore, the luminal epithelium in the cornua and isthmus regions escape desquamation and additionally contribute to re-epithelialization. By day 4, two-thirds of the endometrium lining is covered by epithelium and re-epithelialization is completed by day 6 [183].

Endometrial regeneration essentially includes four important events: (i) proliferation and migration of residual glandular and luminal epithelial cells with the aim to re-epithelialize the lumen during the process of repair; (ii) cellular transdifferentiation of stromal cells into epithelial cells, an event called mesenchymal to epithelial (MET) transition; (iii) engraftment of bone marrow cells into the endometrium and (iv) contribution of progenitor stem cells to a more differentiated progeny [184,185]. The repair of endometrium occurs when circulating E2 levels are still low and epithelial cells lack ER-α in a rapid scar-free process, complete within 48 h, highlighting the conserved wound healing mechanism in the endometrium [186].

It is a mystery how residual glandular epithelial cells proliferate in the absence of hormones while the mechanism underlying their migration to the luminal epithelium is also poorly understood. A role of growth factors including EGF and hepatocyte growth factor (HGF) in the mediation of glandular cell migration has been hypothesized [187,188]. Other “wound-healing” factors such as Activin A, VEGF, cysteine-rich secretory protein 3 (CRISP3), and galectin-7 (GAL-7), as well as the activation of development pathways including WNT/β-catenin and NOTCH are thought to contribute to re-epithelialization and endometrial wound repair [189,190,191,192,193,194]. However, these studies require further consolidation. Androgen receptor (AR) signaling has been recently proposed as a potential regulator of endometrial wound repair in mice and further studies are underway to address the underlying mechanism [195,196].

Although endometrial inflammation results in tissue breakdown, it is also likely to form a fundamental component of the repair process. Indeed, the recruited leukocytes at the time of menstruation have an active role in the endometrial repair whereas depletion of neutrophils in mice results in a profound impairment of this process [197]. In addition to inflammatory cells, increased chemokine production in the perimenstrual endometrium may itself contribute to the endometrial repair process. IL-8 increases during the late secretory phase under the control of hypoxia inducible factor (HIF)-1α [198,199]. Endometrial expression of connective tissue growth factor (CTGF) is also increased in the repairing endometrium and at sites of connective tissue formation under the influence of PGF2a [200,201,202]. Lastly, platelet-rich plasma (PRP) was recently documented to facilitate endometrial repair [203]]. Platelets contain granules rich in growth factors and cytokines including VEGF, TGFβ, PDGF, FGF, IGF1, EFG, HGF, CXCL12, and CCL5. These are released in response to platelet activation at the site of inflammation, in this case endometrial wound, where they activate stromal cells and recruit leukocytes to promote angiogenesis and induce repair mediated by cell proliferation and migration. These platelet-derived factors are pivotal to endometrial progenitor cell activity [204]. Several types of endometrial stem/progenitor cells are present in the endometrium including mesenchymal stem cells (eMSCs), epithelial progenitor cells (eEPs), and side population (SP) cells [186]. Although a number of markers have been identified for the recognition and isolation of these populations, their exact roles in endometrial regeneration is unclear. It is suggested that eEPs are located in the base of the glands and are the source of the proliferative cells for re-epithelialization [205]. A recent study has proposed that endometrial stem cells can promote the repair of stromal cells by activating the p38 MAPK and Akt signaling pathways [206]. Deep sequencing and epigenetic profiling of endometrial stem/progenitor cells and their differentiated progeny will shed new light on their regulations and functions. It would be interesting to examine whether these stem cells participate in the process of MET during regeneration [207]. EVs have been proposed to mediate endometrial and progenitor cell deposition to the endometrial surface to contribute to re-epithelization. In this hypothesis, after endometrial shedding, the platelets released in the uterine cavity initially secrete soluble factors to mobilize cells towards the surface and then export vesicles to commit cells to re-epithelization [208]. Characterization of the EV-cargo and the mechanism underlying their internalization from endometrial cells will consolidate the aforementioned newly developed hypothesis.

Following re-epithelization, local E2 availability increases and orchestrates endometrial epithelial and stromal cell growth and proliferation, which is associated with profound angiogenesis.

## 8. Angiogenesis Route: Building the Endometrial Vascular Network

Formation of new blood vessels from already existing capillaries defines angiogenesis, a two-step process essential for endometrial function [209]. Blood vessels must be repaired during the end of the menses and then capillaries need to grow, mature, and coil during the proliferative and secretory phase. It is accepted that vessel growth in human endometrium occurs by a nonsprouting elongation in response to metabolic demands of surrounding cells and intense hypoxia in the luminal portion of the endometrium [210,211]. The absence of sprouting elongation is in line with the lack of ERα endothelial receptor and, hence, no active proliferation cascades [212]. Endothelial cells forming the capillary bed are under the influence of factors produced by surrounding tissue and angiogenic factors that circulate in the blood during the menstrual cycle. Vascular repair and angiogenesis in the endometrium are dominated by local hypoxia and nER signaling during the follicular phase of the cycle, but vascular maturation occurs during the secretory phase under P4 influence. While hypoxia is a major regulator of endometrial remodeling during menstruation, E2 plays an important role in the reconstruction of a new vascular network and rapid vessel growth [213]. VEGF governs human angiogenesis with the help of two tyrosine kinase receptors, VEGFR-1 and VEGFR-2 [214,215]. Most biological effects of VEGF are mediated by VEGFR-2 [180]. The expression of VEGF in the human endometrium is well described and its involvement in endothelial cell proliferation, migration and assembly of capillary tubes is well documented [216]. However, VEGF is also an essential factor for the first wave of angiogenesis occurring during repair and possibly plays an important role in re-epithelialization [217]. Hypoxia is a known inducer of VEGF via activation of HIF-1α in human endometrial stromal cell, which is suppressed under normoxic conditions [218]. In the glandular and stromal endometrial cells, HIF-1α is abundant during the late secretory phase and menstruation, thus appearing to be related to the process of menstruation [219]. HIF-1α binds directly to the hypoxia-response elements (HRE) in the promoters of the genes encoding VEGF [220]. Activation of nER can also induce VEGF in cultured endometrial stromal cells while nPR signaling inhibits its transcription [221]. The P4-inhibition of VEGF is potentially indirectly mediated by the nPR-induced downregulation of the nER in the human endometrium. Angiopoietins (ANGPT) comprise a second key group facilitating angiogenesis with roles in the regulation of vessel growth, maturation and regression with interesting interactions with VEGF [222]. ANGPT1 promotes the association of endothelial cells with pericytes and vascular smooth muscle cells, which contributes to the maturation of newly formed blood [223]. In the presence of VEGF, a natural antagonist of ANGPT1, ANGPT2 initiates neovascularization. The balance in the availability of ANGPT1 and ANGPT2 is critical for angiogenesis [224]. Concurrently with VEGF induction, hypoxia increases ANGPT2/ANGPT1 ratio, which is associated with new blood vessel formation [180]. E2 also increases ANGPT2/ANGPT1 ratio by decreasing the expression of ANGPT1 [225]. The increase in the ANGPT2/ANGPT1 ratio and VEGF creates an optimum environment for the development, reconstruction and remodeling of the endometrial blood vessels. A lower ANGPT2/ANGPT1 ratio, following exposure of stromal cells to P4, appears to favor the maturation and stabilization of the newly developed vessels in the endometrium, which may underlie angiogenic actions during secretory phase. Another commonly known mediator of angiogenesis in the endometrium is the stromal cell-derived factor 1 (SDF-1), a member of the CXC chemokine family, that signals through its only receptor CXCR4 [226]. SDF-1 and VEGF interact to promote changes in gene expression in relation to angiogenesis [227]. Surprisingly, hypoxia decreases the expression and synthesis of SDF-1, in contrast to nER signaling [228]. The mechanism of this inhibition may be facilitated by two hypoxia-induced transcription factors: the activator protein 1 (AP-1) and NF-κB [229]. Expectedly, P4 antagonizes the E2-stimulation of SDF-1, hence steroid hormones rather than hypoxia may be the main regulator for SDF-1 [230]. Angiogenin (ANG) is another potent inducer of angiogenesis operating under the influence of hypoxia, which induces its expression in stromal and epithelial endometrial cells [231]. However, the major suppliers of ANG in the endometrium undergoing angiogenesis are thought to be infiltrating leukocytes such as natural killer cells and decidual macrophages during postpartum involution [232,233]. Considering that imbalanced expression of ANG has been associated with multiple pathologies including endometriosis, more research is pending to understand the mechanism of its regulation in the endometrium [234].

The previously described effects of E2 are exerted via ERα either directly or indirectly, acting on endometrial epithelial and stromal cells to secrete angiogenic growth factors [235]. Instead, E2 signaling is believed to be mediated by ERβ in endometrial endothelial cells [212]. Still some controversy exists as to which ER operates the angiogenesis specific activities in the endometrial cell subtypes throughout the cycle [236]. There are also conflicting reports regarding the presence of PR in the vascular endothelium [237,238].

Participation of WNT proteins in the process of vasculogenesis and angiogenesis is described [44]. A sustained WNT pathway activation can be utilized to generate endothelial progenitors from mesodermal lineage of embryonic stem cells. The WNT5A ligand is a potential protagonist in endothelium recovery resulting in angiogenesis, as it takes part in the healing of the damaged endothelium, but not in proliferation and migration of the endothelial cells nor elongation [239,240]. WNT7A of epithelial origin might be a chemoattractant for endothelial cells in the process of physiological endometrial angiogenesis and it is upregulated during the proliferative phase [193]. Finally, β-catenin can function in the endometrium either directly on endothelial cells or indirectly through its action on endometrial cells where it promotes the expression of VEGF [237]. WNT/β-catenin pathway in the process of endometrial angiogenesis is still largely unexplored.

## 9. Exit

The ability of endometrial cells to perceive and correctly respond to their microenvironment forms the basis of homeostasis. Errors in endometrial cell signaling interactions and cellular information processing are responsible for endometrial disease that can span in severity from poor endometrial receptivity to cancer. Analysis of endometrial cell signaling networks with a combination of experimental and theoretical approaches, including modeling and simulation, has been informing the scientific community over the years. Yet, persistent gaps do not allow for the synthesis of the complete physiological endometrial signaling landscape. An in-depth appreciation of the hitherto literature identifies the WNT pathway as a contributor to the gaps responsible for the incomprehension of various signaling “routes” of the endometrial cell “tube map”. The all-pervasive WNT signaling pathway exploits various molecular regulators of endometrial cycling, standing up to its sobriquet as the “chameleon” of the physiological endometrial signaling. Novel trajectories of endometrial cell signaling should be explored considering recent discoveries of diverse embryo-endometrial communication mechanisms utilizing EVs [241] and the contribution of stem cells to endometrial pathophysiology [186]. However, revisiting previously performed global expression studies and utilizing meta-analyses to screen out inconsistencies and clarify interpretations is additionally a valuable strategy to contribute to the state-of-the art.

The meticulous study of endometrial signaling pathways potentiates both the discovery of novel therapeutic targets to tackle disease and the development of artificial endometrium, a staple tissue for futuristic in vitro gestations.
